# Population Preferences for Primary Care Models for Hypertension in Karnataka, India

**DOI:** 10.1001/jamanetworkopen.2023.2937

**Published:** 2023-03-14

**Authors:** Hannah H. Leslie, Giridhara R. Babu, Nolita Dolcy Saldanha, Anne-Marie Turcotte-Tremblay, Deepa Ravi, Neena R. Kapoor, Suresh S. Shapeti, Dorairaj Prabhakaran, Margaret E. Kruk

**Affiliations:** 1 Division of Prevention Science, Department of Medicine, University of California, San Francisco, San Francisco; 2 Department of Global Health and Population, Harvard T.H. Chan School of Public Health, Boston, Massachusetts; 3 Indian Institute of Public Health–Bangalore, Public Health Foundation of India, Bengaluru, Karnataka; 4 VITAM–Laval University Sustainable Health Research Center, Quebec City, Quebec, Canada; 5 Faculty of Nursing, Laval University, Quebec City, Quebec, Canada; 6 Centre for Chronic Conditions and Injuries, Public Health Foundation of India, New Delhi

## Abstract

**Importance:**

Hypertension contributes to more than 1.6 million deaths annually in India, with many individuals being unaware they have the condition or receiving inadequate treatment. Policy initiatives to strengthen disease detection and management through primary care services in India are not currently informed by population preferences.

**Objective:**

To quantify population preferences for attributes of public primary care services for hypertension.

**Design, Setting, and Participants:**

This cross-sectional study involved administration of a household survey to a population-based sample of adults with hypertension in the Bengaluru Nagara district (Bengaluru City; urban setting) and the Kolar district (rural setting) in the state of Karnataka, India, from June 22 to July 27, 2021. A discrete choice experiment was designed in which participants selected preferred primary care clinic attributes from hypothetical alternatives. Eligible participants were 30 years or older with a previous diagnosis of hypertension or with measured diastolic blood pressure of 90 mm Hg or higher or systolic blood pressure of 140 mm Hg or higher. A total of 1422 of 1927 individuals (73.8%) consented to receive initial screening, and 1150 (80.9%) were eligible for participation, with 1085 (94.3%) of those eligible completing the survey.

**Main Outcomes and Measures:**

Relative preference for health care service attributes and preference class derived from respondents selecting a preferred clinic scenario from 8 sets of hypothetical comparisons based on wait time, staff courtesy, clinician type, carefulness of clinical assessment, and availability of free medication.

**Results:**

Among 1085 adult respondents with hypertension, the mean (SD) age was 54.4 (11.2) years; 573 participants (52.8%) identified as female, and 918 (84.6%) had a previous diagnosis of hypertension. Overall preferences were for careful clinical assessment and consistent availability of free medication; 3 of 5 latent classes prioritized 1 or both of these attributes, accounting for 85.1% of all respondents. However, the largest class (52.4% of respondents) had weak preferences distributed across all attributes (largest relative utility for careful clinical assessment: β = 0.13; 95% CI, 0.06-0.20; 36.4% preference share). Two small classes had strong preferences; 1 class (5.4% of respondents) prioritized shorter wait time (85.1% preference share; utility, β = −3.04; 95% CI, −4.94 to −1.14); the posterior probability of membership in this class was higher among urban vs rural respondents (mean [SD], 0.09 [0.26] vs 0.02 [0.13]). The other class (9.5% of respondents) prioritized seeing a physician (the term *doctor* was used in the survey) rather than a nurse (66.2% preference share; utility, β = 4.01; 95% CI, 2.76-5.25); the posterior probability of membership in this class was greater among rural vs urban respondents (mean [SD], 0.17 [0.35] vs 0.02 [0.10]).

**Conclusions and Relevance:**

In this study, stated population preferences suggested that consistent medication availability and quality of clinical assessment should be prioritized in primary care services in Karnataka, India. The heterogeneity observed in population preferences supports considering additional models of care, such as fast-track medication dispensing to reduce wait times in urban settings and physician-led services in rural areas.

## Key Points

**Question:**

How can government health care services best meet population preferences for ongoing hypertension treatment in Karnataka, India?

**Findings:**

In this cross-sectional study of 1085 adults with hypertension, a discrete choice experiment revealed that 85% of respondents prioritized careful clinical assessment and consistent availability of free medication. Some urban respondents prioritized shorter wait times, and some rural respondents prioritized seeing a physician vs a nurse.

**Meaning:**

The population preferences identified in this study suggest that consistent medication availability and quality of clinical assessment are key priorities for strengthening primary care services for adults with hypertension in urban and rural areas of Karnataka, India.

## Teaser

This cross-sectional study uses household surveys and a discrete choice experiment to assess preferences for primary care services among adults with hypertension in Karnataka, India.

## Introduction

Cardiovascular disease accounts for more than 28% of total deaths and 25% of years of life lost among adults older than 50 years in India,^1,2^ with hypertension affecting more than 200 million individuals and playing a role in at least 1.6 million deaths annually.^3,4^ In Karnataka state, where hypertension prevalence exceeds 20%,^5^ awareness of disease status is low. Although more than 80% of those diagnosed with hypertension have initiated treatment, only 62% of those receiving treatment have reported consistent medication use.^4,6,7^

Population-level hypertension control depends on effective primary care.^8^ Indian policy has prioritized prevention and control of noncommunicable diseases through primary care services since implementation of the 2010 National Programme for Prevention and Control of Cancer, Diabetes, Cardiovascular Diseases and Stroke.^9,10^ The focus on prevention and control of noncommunicable diseases was further emphasized in the 2018 National Health Protection Mission, which included the planned provision of comprehensive primary care services through 150 000 new health and wellness centers (HWCs).^11,12,13,14^ However, the extensive primary care system has faced challenges in adapting to the changing needs and preferences of an increasingly educated and rapidly urbanizing population.^15,16,17,18,19,20^ Disease burden, availability of health care services, and service use patterns differ between rural and urban areas.^19,21,22^ Only in the past decade has policy begun to be standardized instead of being administered through separate rural and urban national health missions, and differences persist in services and health care use. Private care services are concentrated in urban areas, and public community health centers (CHCs) are predominantly located in rural areas.^7^ While the bypassing of public primary care has been common across all households with members diagnosed with hypertension, urban respondents have been less likely to use public primary care services than rural respondents (11% vs 23%, respectively).^23^

Discrete choice experiments (DCEs) are a method of quantifying population preferences to inform health care service delivery.^24,25,26,27,28,29^ We conducted a DCE to characterize stated preferences for key aspects of health care services among adults with hypertension in an urban district and a rural district in Karnataka, India.

## Methods

This cross-sectional study was reviewed and approved by the Harvard Human Research Protection Program, the Public Health Foundation of India Institutional Ethics Committee, and the Indian Institute of Public Health–Bengaluru Campus Institutional Ethics Committee. Permission for study procedures was received from the Indian Council of Medical Research Health Ministry Screening Committee, the Karnataka State Directorate of Health and Family Welfare, and the chief health officers and chief medical officers in participating districts. All participants provided written informed consent. This study followed the Strengthening the Reporting of Observational Studies in Epidemiology (STROBE) reporting guideline for cross-sectional studies. The study reflexivity statement is available in eTable 1 in Supplement 1.

### Study Setting

The state of Karnataka, India, is home to more than 66.8 million people, with 39% residing in urban areas.^30^ This study was conducted in the Bengaluru Nagara district (Bangalore City; 4400 residents per km^2^, with approximately 10 million total residents) and the Kolar district (384 residents per km^2^, with approximately 1.5 million total residents). Districts were selected to provide urban (Bengaluru Nagara) and rural (Kolar) study sites that (1) were accessible to the Bangalore-based study team throughout the period of COVID-19–related restrictions, (2) were located in areas in which district health officials were receptive, and (3) had an HWC program that was initiated but not fully operational. Data collection took place from February 2 to February 21, 2021, for the DCE development phase and from June 22 to July 27, 2021, for administration of the household surveys that included the DCE.

### Development of Discrete Choice Experiment

This study aimed to assess how government health care services could best meet population preferences for ongoing hypertension treatment. The DCE was designed in accordance with the checklist developed by the Good Research Practices for Conjoint Analysis Task Force^31^ (full details are provided in eMethods in Supplement 1). In brief, consistent with recommended practice,^31^ we reviewed published literature and national and state policy to identify an extensive list of attributes that were relevant to ongoing policy innovations in primary care, amenable to intervention at the district health care system level, and pertinent to individuals with hypertension.^31,32,33,34^ We then conducted 6 focus groups in both urban and rural settings; each focus group comprised 5 to 7 patients receiving care for hypertension to elicit perspectives on their experiences and the relevance of these attributes. The focus groups enabled participants to identify relevant attributes that were not part of the initial list derived from the literature and expert consultations, to rank their own priorities among the proposed attributes, and to suggest attribute levels based on their own experiences. The research team (H.H.L., G.R.B., N.D.S., A.T.T., S.S.S., D.P., and M.E.K.) synthesized focus group findings to select the final attributes and levels (Table 1) to include in the DCE based on priority among patients, relevance to the research question, and independence across attributes.

**Table 1.** Attributes and Levels of the Discrete Choice Experiment

Attribute	Levels
Staff attitudes, including nonclinical personnel such as security	Clinic staff members are courteous
	Clinic staff members are not always courteous
Total wait time	15 min
	30 min
	1 h
	2 h
	3 h
	5 h
Clinician type	Physician^a^
	Nurse
Quality of clinical assessment	Medical staff assess patients carefully
	Medical staff do not always assess patients carefully
Availability of free medication	Free medication is available in this facility
	Free medication is not always available in this facility

^a^ The term *doctor* was used in the survey.

### Experimental Design

We designed the DCE with 2 alternatives per choice set and no opt-out option to ensure responses would be provided by all participants. The DCE scenario alternatives were selected to ensure balance and optimize determinant efficiency (a measure of the goodness of a design relative to a hypothetical orthogonal design); 35 choice sets were generated in 5 versions. A choice with 1 option designed to emulate the HWC was added to all versions, resulting in 8 choice sets per respondent (the introductory script and an example of a choice card are available in eFigure 1 in Supplement 1). Data collectors selected 1 version for each respondent, cycling through the 5 versions in turn. Additional survey items addressed demographic characteristics, health status, and previous use of and perspectives on health care services. To ensure consistency, all survey materials, which were originally written in English, were translated into Kannada, then translated back to English by the research team (G.R.B., N.D.S., and D.R.); respondents could select English or Kannada. We pretested all survey materials among 10 respondents.

### Sample Size

We calculated the target sample size based on the power required to maximize the efficiency of the DCE. To calculate the minimum sample size, the largest number of levels for an attribute (including interaction terms) was divided by the product of the number of alternatives in each task multiplied by the number of tasks; this quotient was then multiplied by 500.^35,36^ We calculated the minimum sample size before finalizing the DCE design, using 2 as the number of alternatives in each task, 8 as the number of tasks, and 10 as the product of the largest number of levels of an attribute (5) and a binary interaction term, such as study site or a 2-level attribute. This calculation yielded a minimum of 312 participants per subgroup. To ensure a robust sample size for estimation and enhance the generalizability of findings, we targeted 500 participants per location.

### Sampling

We used maps of administrative areas created for the most recent National Health Mission immunization campaign as a sampling framework.^37^ In the Bengaluru Nagara district, we identified 2 wards (among 198 total wards; mean population, 42 500 per ward) that included both informal settlements and formal areas and could be accessed by the research team. Each ward was divided into approximately 20 units comparable with a city block. The Kolar district includes 6 administrative subdivisions; we selected the main Kolar area, which includes the largest number of village clusters, to represent the rural setting of the district, and we selected 2 of 343 villages using convenience sampling. Field teams visited each unit or village and used systematic random sampling of households until the target of 500 surveys per district was reached.

### Survey Administration

Eligibility assessment included a brief informed consent process, a questionnaire, and hypertension screening in accordance with Indian national guidelines. Two blood pressure (BP) measures were obtained (with a third obtained if the difference between the first and second measurements was >5 mm Hg for either systolic or diastolic BP), and the lower BP value was used. Elevated BP was defined as systolic BP of 140 mm Hg or higher or diastolic BP of 90 mm Hg or higher. Eligibility was assessed based on age (≥30 years) and self-reported diagnosis of hypertension and/or elevated BP at the time of the survey; pregnant women were ineligible. All individuals with elevated BP and no previous diagnosis of hypertension received information on government-approved nearby sources of care regardless of study participation. In Bengaluru Nagara, interested adult residents in the households received screening, and 1 resident was selected from those eligible. In Kolar, a Kish grid^38^ was used to select adults in random order for screening. Eligible adults were invited to participate in the full study after providing a second consent; only 1 respondent was enrolled per household.

### Statistical Analysis

Data were collected on tablet computers using the Research Electronic Data Capture (REDCap) database and synced to servers daily. Stata software, version 17.0 (StataCorp LLC), was used for data cleaning and analysis, with additional packages including dcreate, mixlogit, and lclogit2.^39,40,41,42^ We used R software, version 4.2.1 (R Foundation for Statistical Computing), with packages dplyr, foresplot, tidyverse, readxl, stringr, and haven for the forest plot.

Descriptive statistics were used to summarize the demographic characteristics (including gender, age, caste, educational level, and occupation) of the study population; information was collected on caste rather than race and ethnicity in accordance with the Census of India and all major population-based surveys. We conducted robustness checks (details are available in eMethods in Supplement 1) and then fit mixed logit models. These models estimated the likelihood of selecting a clinic as a function of clinic attributes; parameters were allowed to vary randomly across individuals to account for heterogeneity in preferences and scale and to address nonindependence of multiple responses within individuals. The results provided estimates of the mean relative utility of each attribute level within the DCE as well as the SD of the estimated utility. Estimated SDs could be compared with a null hypothesis of 0 variance; direction of the estimate was irrelevant.^39^ Mean relative utility and SDs were considered significant if their 95% CIs excluded the null. After model testing, we fit the full population mixed logit model on all data with normally distributed parameters, independent covariance structure, robust SEs, and 500 Halton draws.

To identify groupings of respondent preferences, we fit a latent class model with up to 8 latent classes^41,43^; we selected the final model based on bayesian information criterion statistics. We estimated posterior probabilities of class membership for each respondent. We calculated preference shares as the percentage of utility for each attribute among the total utility, multiplying utility for wait time by the defined range for this attribute (4.75 hours). We estimated uptake for each latent class comparing a baseline scenario (staff not always courteous, wait time is 3 hours, primary care clinician is a nurse, clinical assessment not always careful, and free medication not always available) with each of the following 3 scenarios: (1) primary care clinician is a physician (the term *doctor* was used in the survey) and other attribute levels the same as baseline (physician-led model), (2) wait time is 30 minutes and other attribute levels are the same as baseline (fast-track model), and (3) clinical assessment always careful and free medication always available and other attribute levels are the same as baseline (based on HWC model). We assessed the association of the observed characteristics of location (Bengaluru Nagara or Kolar), gender (identification as female vs male or other), formal education (none vs primary school, secondary school, or college or higher), and awareness of hypertension diagnosis (yes vs no) with the posterior probability of class membership. Findings for location were reported from unadjusted models; adjusted models including location could not be estimated due to the magnitude of class share differences by location.

## Results

Of 1927 individuals approached, 1422 (73.8%) consented to receive initial screening. Among those who received screening, 1150 (80.9%) were eligible for study inclusion; of those, 1085 individuals (94.3%) consented to and completed the full survey (eFigure 2 in Supplement 1). The mean (SD) age of respondents was 54.4 (11.2) years; 573 (52.8%) identified as female, 507 (46.7%) identified as male, and 5 (0.5%) identified as other genders (Table 2). A total of 530 respondents were from the Bengaluru Negara district, and 555 were from the Kolar district. The majority of respondents (918 [84.6%]; 510 [96.2%] in Bengaluru Nagara and 408 [73.5%] in Kolar) reported being previously diagnosed with hypertension; of those, nearly all respondents (883 of 913 [96.2%]; 492 of 510 [96.5%] in Bengaluru Nagara and 391 of 408 [95.8%] in Kolar) had previously received treatment for hypertension. Government facilities were the most common source of hypertension care in both Bengaluru Nagara (407 of 510 respondents [79.8%]) and Kolar (347 of 408 respondents [85.0%]).

**Table 2.** Respondent Demographic Characteristics by Study District

Characteristic	Respondents, No. (%)		
	Total (N = 1085)	Bengaluru Nagara (n = 530)	Kolar (n = 555)
Age, mean (SD), y	54.4 (11.2)	57.2 (9.6)	51.8 (12.1)
Gender			
Female	573 (52.8)	277 (52.3)	296 (53.3)
Male	507 (46.7)	251 (47.4)	256 (46.1)
Other^a^	5 (0.5)	2 (0.4)	3 (0.5)
Caste^b^			
General category	135 (12.4)	67 (12.6)	68 (12.3)
Scheduled caste or tribe	492 (45.3)	259 (48.9)	233 (42.0)
Other backward caste	262 (24.1)	124 (23.4)	138 (24.9)
Other^c^	191 (17.6)	76 (14.3)	115 (20.7)
Missing	5 (0.5)	4 (0.8)	1 (0.2)
Educational level			
No formal education	261 (24.1)	125 (23.6)	136 (24.5)
Primary school	478 (44.1)	255 (48.1)	223 (40.2)
Secondary school	221 (20.4)	103 (19.4)	118 (21.3)
College or higher	125 (11.5)	47 (8.9)	78 (14.1)
Occupation			
Not employed outside of home	328 (30.2)	247 (46.6)	81 (14.6)
Semiskilled or unskilled	444 (40.9)	107 (20.2)	337 (60.7)
Skilled	296 (27.3)	163 (30.8)	133 (24.0)
Professional	4 (0.4)	2 (0.4)	2 (0.4)
Missing	13 (1.2)	11 (2.1)	2 (0.4)
Ever previously diagnosed with hypertension			
No	167 (15.4)	20 (3.8)	147 (26.5)
Yes	918 (84.6)	510 (96.2)	408 (73.5)
Received medication for hypertension			
No	35 (3.8)	18 (3.5)	17 (4.2)
Yes	883 (96.2)	492 (96.5)	391 (95.8)
Source of hypertension care			
Government facility: primary	419 (45.6)	207 (40.6)	212 (52.0)
Government facility: secondary	335 (36.5)	200 (39.2)	135 (33.1)
Private facility	151 (16.4)	98 (19.2)	53 (13.0)
Other^d^	13 (1.4)	5 (1.0)	8 (2.0)

^a^ Other genders were not specified.

^b^ Caste names and definitions follow the categorization scheme of the Indian national government.

^c^ Other castes were not specified.

^d^ Other includes respondents selecting the option “AAYUSH hospital, NGO at trust hospital” or the option “ASHA (accredited social health activist)/ANM (auxiliary nurse midwife)/MLHP (mid-level health provider).” This includes visits in the community.

The 1085 respondents completed 8656 choice tasks within the DCE. Data quality checks identified no concerns with DCE administration or responses. Details on the validity checks and model fitting are provided in the eMethods in Supplement 1.

Five attributes were included in the DCE: staff attitudes, total wait time, clinician type, quality of clinical assessment, and availability of free medication. The mixed logit model for the full study population (Table 3) revealed that respondents did not highly value courtesy relative to other attributes (utility, β = 0.04; 95% CI, −0.02 to 0.11), were weakly averse to longer wait times (utility, β = −0.09; 95% CI, −0.12 to −0.05), and preferred being seen by physicians over nurses (utility, β = 0.34; 95% CI, 0.24-0.43). The strongest preferences were for careful clinical assessment (utility, β = 0.67; 95% CI, 0.56-0.78) and availability of free medication (utility, β = 0.68; 95% CI, 0.57-0.80). The SDs revealed population heterogeneity in preferences for all attributes except courtesy.

**Table 3.** Results of Mixed Logit Model Assessing Study Population Preferences for Hypertension Care Services

Attribute (N = 1085)^a^	Utility, β (95% CI)
**Mean**	
Total wait time	−0.09 (−0.12 to −0.05)
Clinic staff members are courteous (vs not always courteous)	0.04 (−0.02 to 0.11)
Seen by a physician (vs a nurse)^b^	0.34 (0.24 to 0.43)
Clinicians assess patients carefully (vs do not always assess patients carefully)	0.67 (0.56 to 0.78)
Free medication is available in this facility (vs not always available)	0.68 (0.57 to 0.80)
**SD**	
Total wait time	0.26 (0.20 to 0.32)
Clinic staff members are courteous (vs not always courteous)	0.02 (−0.18 to 0.22)
Seen by a physician (vs a nurse)^b^	0.96 (0.83 to 1.10)
Clinicians assess patients carefully (vs do not always assess patients carefully)	1.24 (1.10 to 1.37)
Free medication is available in this facility (vs not always available)	1.25 (1.11 to 1.39)

^a^ 8656 Choices.

^b^ The term *doctor* was used in the survey.

**Figure 1.**
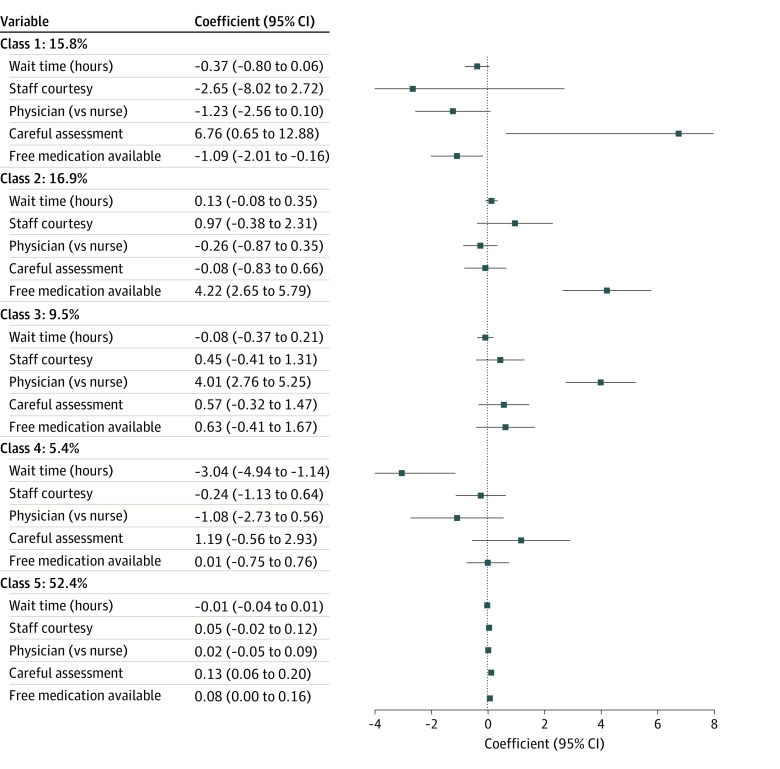
Latent Class Preferences for Hypertension Services

Latent class analysis revealed a 5-class solution optimized model fit. Class 5 members (52.4% of respondents) had significant preferences for careful clinical assessment (utility, β = 0.13; 95% CI, 0.06-0.20) and availability of free medication (utility, β = 0.08; 95% CI, 0.00-0.16), although relative preferences were weakly differentiated (Figure 1). Class 2 members (16.9% of respondents) strongly prioritized availability of free medication (utility, β = 4.22; 95% CI, 2.65-5.79), while class 1 members (15.8% of respondents) prioritized careful clinical assessment (utility, β = 6.76; 95% CI, 0.65-12.88) and had a negative preference for availability of free medication (utility, β = −1.09; 95% CI, −2.01 to −0.16) relative to other attributes. Together, these 3 classes with relative preferences for careful clinical assessment and/or availability of free medication composed 85.1% of the total population. Respondents in the remaining classes had strong preferences for being seen by a physician rather than a nurse (class 3 [9.5% of respondents]; utility, β = 4.01; 95% CI, 2.76-5.25) and for shorter wait time (class 4 [5.4% of respondents]; utility, β = −3.04; 95% CI, −4.94 to −1.14). In each of the 4 smaller classes, the most preferred attribute within the class comprised at least 50% of total utility (50.1% for careful clinical assessment in class 1, 68.5% for availability of free medication in class 2, 66.2% for seeing a physician vs a nurse in class 3, and 85.1% for wait time in class 4) (eFigure 3 in Supplement 1).

**Figure 2.**
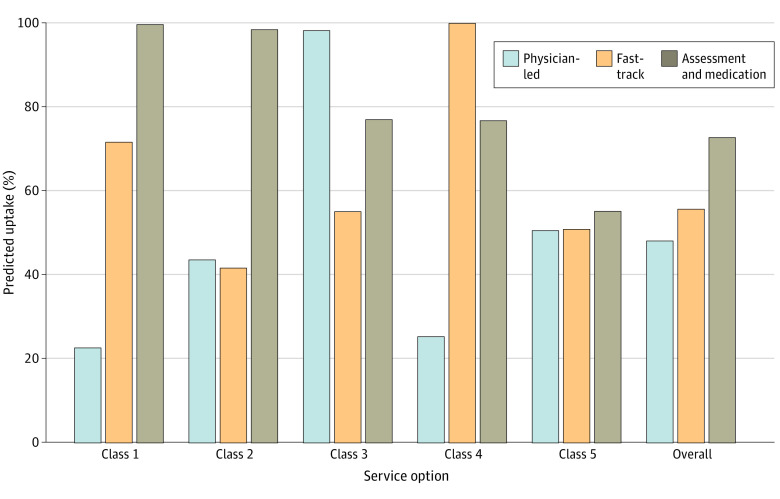
Estimated Uptake of 3 Service Options by Latent Class Each service option was compared with the baseline scenario, which consisted of the following attributes: staff not always courteous, 3-hour wait time, seen by a nurse, examination not always careful, and free medication not always available. Uptake of 50% represents uptake equivalent to that of the baseline service option. Physician-led indicates a scenario in which the individual is seen by a physician, with other attribute levels unchanged from the baseline scenario. Fast track describes a scenario with 30-minute wait time, with other attribute levels unchanged from the baseline scenario. Assessment and medication indicates a scenario in which clinical assessment is always careful and free medication is always available, with other attribute levels unchanged from the baseline scenario.

The estimated uptake of services in the scenario in which preferences for careful clinical assessment and consistent availability of free medication were met relative to the baseline scenario of low-quality care was high overall (72.7%) and within each class (99.7% in class 1, 98.4% in class 2, 77.0% in class 3, and 76.8% in class 4), with the exception of class 5 (55.2%), which had weak relative preferences that yielded little difference in estimated uptake across scenarios (Figure 2). Because class 5 comprised approximately one-half (52.4%) of the study population, weak uptake within this class constrained overall uptake estimates. In the smaller classes that prioritized seeing a physician (class 3) or shorter wait time (class 4), meeting these preferences increased the estimated uptake even more (98.2% for class 3 under the physician-led scenario and 99.9% for class 4 under the 30-minute wait time scenario) than the scenario in which preferences for careful clinical assessment and consistent availability of free medication were met.

Class membership differed by location (eTable 2 in Supplement 1), with 3 classes having strong divergence between sites. The mean (SD) posterior probabilities of being in class 1 (which prioritized careful clinical assessment) were 0.28 (0.44) in Kolar vs 0.03 (0.13) in Bengaluru Nagara, and the mean (SD) posterior probabilities of being in class 3 (which prioritized seeing a physician) were 0.17 (0.35) in Kolar vs 0.02 (0.10) in Bengaluru Nagara. Conversely, the mean (SD) share of respondents prioritizing wait time (class 4) was 0.09 (0.26) in Bengaluru Nagara and only 0.02 (0.13) in Kolar. In a model adjusted for gender, level of formal education, and knowledge of hypertension diagnosis (eTable 3 in Supplement 1), individuals without formal education had higher odds of belonging to class 2 (which prioritized availability of free medication; adjusted odds ratio [aOR], 1.88; 95% CI, 1.27-2.77) or class 4 (which prioritized shorter wait time; aOR, 2.22; 95% CI, 1.19-4.13) than belonging to class 5 (which had weak relative preferences). Individuals unaware of their hypertension status had higher odds of belonging to classes prioritizing careful clinical assessment (class 1: aOR, 3.54; 95% CI, 2.21-5.66) or seeing a physician (class 3: aOR, 4.01; 95% CI, 2.34-6.87) than belonging to class 5.

## Discussion

This cross-sectional study of population preferences for hypertension care services in Karnataka, India, found overall preferences for careful clinical assessment and/or consistent availability of free medication among the majority of respondents. Approximately one-half (52.4%) of respondents had only weak relative preferences for these attributes. Additional smaller preference classes included primarily rural respondents who prioritized seeing a physician rather than a nurse and primarily urban respondents who prioritized shorter wait times. Given these findings, addressing medication availability and ensuring clinical competence are key priorities in the continued expansion of the HWC primary care model.

Previous analyses of national surveys and qualitative studies^20,23,44,45,46^ found that patients cited long wait times and lack of medication and diagnostic assessment as well as inconsistent clinician availability as major concerns with public primary care clinics. Our study refined this understanding to quantify relative preferences, finding that for most respondents, longer wait times might be acceptable if stronger preferences for consistent medication availability and/or competent care were met. The findings highlighted the importance of resolving supply chain issues that play a role in the inconsistent availability of medications^47^ and focusing attention on the need for a competent workforce that has time to provide careful assessments.^17^ Notably, most respondents were willing to be seen by nurses if other preferences were met; only a small proportion of respondents (9.5%), primarily in the rural setting, prioritized physician-led care. While not directly comparable due to differences in DCE design and study population, a DCE among residents of urban slums in Ahmedabad, India, similarly identified competent care as the highest priority overall and found heterogeneity in clinician preference (among traditional, private, and public facilities) by socioeconomic status^32^; we found that availability of free medication and shorter wait times were particularly important for individuals without formal education. Our findings build on previous work in Karnataka, which found that patients were interested in competent care for noncommunicable diseases^48^ and suggested that rapid expansion of the nonphysician HWC model in Karnataka may be an appropriate approach to alleviate the overload placed on primary care physicians.^18,49^ At the same time, physician-led care will remain important for diagnoses and prescription treatment; legally, only physicians can prescribe medication or change treatment regimens, while nurses can manage ongoing treatment. We did not find that courteous treatment was highly valued relative to other attributes, despite the experiences of disrespectful care reported during the focus groups for development of this DCE and in other studies.^32,48^ This finding may reflect respondents’ willingness to trade discourteous treatment for more competent care or more convenient services, particularly given that most respondents had experience with hypertension treatment. It was also notable that proposed elements of HWC, such as yoga services,^48^ were not prioritized in focus group discussions.

The current cross-sectional study was conducted in a setting of policy innovation in primary care delivery. As of early 2021, more than 2000 HWCs were operational in Karnataka based on data from the state government^49^; per policy, these facilities are staffed by nonphysician clinicians who directly provide screening and ongoing treatment support for hypertension while overseeing lay health care workers who extend screening into the community. As a safeguard, HWCs are supported by physicians with prescription capabilities at referral facilities. In this context, 3 policy implications were identified. First, continued expansion of HWCs should prioritize medication availability and competent care, including care from nonphysicians, in both urban and rural settings as a primary goal for responding to stated population preferences. Second, subsets of the study population expressed preferences consistent with differentiated models of care, such as fast-track and physician-led services. There is a basis for service delivery innovations in Karnataka through efforts such as evening clinics in informal settlements^50^; looking forward, the National Digital Health Mission includes ambitious plans for electronic health records to ensure continuity of care across service locations, which could improve consistent access to medications if fully implemented. Third, monitoring of service use and further research on service uptake would help clinicians and policy makers understand the group of comprehensive decision makers who had weak relative preferences for the attributes assessed in this study.

### Limitations

This study has several limitations. Administrative areas were selected based on the feasibility of conducting research during the period of COVID-19 pandemic restrictions and the provision of support from district health officials; results may not be generalizable to all districts in Karnataka or to other states in India. The respondent population included a high proportion who were aware of their hypertension diagnosis, potentially due to greater interest in study participation among these individuals. The results provide less opportunity to draw inferences on aspects of service delivery that would help to reach and retain those with currently undiagnosed hypertension. We designed a forced-choice DCE, in which respondents could not opt out of the choice task presented, to optimize internal validity; DCE estimates have been found to be accurate among those who are truly likely to use a potential service or product.^29^ This design has less external validity in identifying those who are unlikely to use services overall or to use services based on the attributes and levels studied; our uptake estimates should be interpreted as revealing relative trade-offs rather than absolute estimates of population-level use of primary care services. The DCE findings are applicable to the specific attributes and levels assessed; it is possible that the relatively weaker preferences and lower estimated service uptake among the largest class of respondents reflect preferences not captured in this DCE. Some respondents may have considered the attributes of seeing a physician and medication availability collectively due to current policy restricting the authority to provide prescriptions for medication to physicians only. We worded the attributes to focus on independent aspects of care and encouraged respondents to consider the choices as hypothetical to reduce the possibility of conflating attributes.

## Conclusions

This cross-sectional study involving population preference assessment found that adults with hypertension prioritized consistent medication availability and quality of clinical assessment in both urban and rural settings within Karnataka, India. Evaluation of additional models of care, such as physician-led services and fast-track medication dispensing to reduce wait times, may be warranted to fully address heterogeneity in population preferences.

## Notes

Supplement 1. **eMethods.** Development of Discrete Choice Experiment and Validity Checks and Model Fitting **eTable 1.** Reflexivity Statement **eFigure 1.** Discrete Choice Experiment Introductory Script and Choice Card **eFigure 2.** Study Flowchart **eFigure 3.** Preference Shares for 5 Latent Classes **eTable 2.** Latent Class Shares by Location **eTable 3.** Factors Associated With Latent Class Membership

Supplement 2.
Data Sharing Statement
