# Error in Affiliation

**DOI:** 10.1001/jamanetworkopen.2023.54465

**Published:** 2024-01-12

In the Original Investigation titled “Population Preferences for Primary Care Models for Hypertension in Karnataka, India,”^1^ published March 14, 2023, the affiliation for author Giridhara R. Babu, MBBS, PhD, was incorrect. The affiliation has been amended to read, “Department of Population Medicine, College of Medicine, QU Health, Qatar University, Doha, Qatar.” This article has been corrected.^1^

## References

[1] Leslie HH, Babu GR, Dolcy Saldanha N, et al. Population preferences for primary care models for hypertension in Karnataka, India. *JAMA Netw Open*. 2023;6(3):e232937. doi:10.1001/jamanetworkopen.2023.2937 PMC10015308 36917109

