# Supplementary material for: Population Preferences for Primary Care Models for Hypertension in Karnataka, India
Source: JAMA Netw Open. 2023 Mar 14;6(3):e232937. doi: 10.1001/jamanetworkopen.2023.2937 (PMC10015308; doi:10.1001/jamanetworkopen.2023.2937)
Supplement: Supplement 1. — eMethods. Development of Discrete Choice Experiment and Validity Checks and Model Fitting eTable 1. Reflexivity Statement eFigure 1. Discrete Choice Experiment Introductory Script and Choice Card eFigure 2. Study Flowchart eFigure 3. Preference Shares for 5 Latent Classes eTable 2. Latent Class Shares by Location eTable 3. Factors Associated With Latent Class Membership [file jamanetwopen-e232937-s001.pdf]

Supplementary Online Content

Leslie HH, Babu GR, Dolcy Saldanha N, et al. Population preferences for primary care models for hypertension in Karnataka, India. *JAMA Netw Open*. 2023;6(3):e232937. doi:10.1001/jamanetworkopen.2023.2937

eMethods. Development of Discrete Choice Experiment and Validity Checks and Model Fitting

eTable 1. Reflexivity Statement

eFigure 1. Discrete Choice Experiment Introductory Script and Choice Card

eFigure 2. Study Flowchart

eFigure 3. Preference Shares for 5 Latent Classes

eTable 2. Latent Class Shares by Location

eTable 3. Factors Associated With Latent Class Membership

This supplemental material has been provided by the authors to give readers additional information about their work.

© 2023 Leslie HH et al. *JAMA Network Open*.

eMethods. Development of Discrete Choice Experiment and Validity Checks and Model Fitting

Development of Discrete Choice Experiment

Study plan
We developed a protocol for the DCE development, cross-sectional administration, and analysis and submitted it to the Institutional Ethics Committee of the Indian Institute of Public Health. We adapted the analysis plan to fit the final DCE design – including testing if preferences for wait time followed a linear form – and by including latent class analysis given evidence of heterogeneity in preferences within the study respondents. Research team participation in each aspect of the study is reported in the Reflexivity Statement.

Patient and Public Involvement
Patients receiving care for hypertension were involved in DCE design through the focus groups described below. These perspectives and ranking of potential attributes informed the experimental design of the DCE.

Development of the Discrete Choice Experiment: Qualitative Research
Focus groups included 5-7 adults aged 30 and over currently receiving care for hypertension and were stratified by district and sex; participants were identified using convenience sampling at health centers or door to door with referrals and assistance from Accredited Social Health Activists (a trained community health worker operating as a link between community members and health services). Four groups were conducted in urban Bengaluru and 2 in Kolar based on greater heterogeneity of service options and experiences in the urban setting. All participants provided informed consent; procedures for Covid-19 safety were followed. Trained moderators led discussions around participants' experience initiating and maintaining hypertension treatment, perceptions of quality of care, and preferences for future care. Near the end of discussion, participants were given 20 stickers and asked to assign them to a list of example attribute levels (Table 1) based on how they valued each; the groups then reconvened to discuss the results. This exercise enabled participants to provide input on attributes that were included in local policy but may not have come up during discussions and gave a basis for more detailed discussion of specific attribute levels individuals had experienced. Discussions were conducted in Kannada and were transcribed and translated for review. Two members of the research team (NDS and AMTT) reviewed transcripts to identify themes in patient experience and synthesized the resulting priorities within and across the 6 focus groups; we also tallied rankings across all participants (Table 2).

Table 1: Attributes of potential relevance

Competence and knowledge of providers
Low cost
Shorter travel time
Shorter wait time to see a provider
Availability of health care providers
Timing of clinic hours

© 2023 Leslie HH et al. *JAMA Network Open*.

Availability of medications
Kindness and respect from providers
Availability of all services at the same facility
Ability to receive other non-hypertension services at the same facility
Other counseling (adherence support, psychosocial support)
Peer support services
Integrated care services
Continuity of service providers
Insurance coverage
Incentives (reimbursement for travel, travel voucher, food baskets)

Attributes prioritized during focus group discussions aligned with levels prioritized in the sticker ranking process, with additional insight around the issue of medication, where respondents identified the cost of medication in private facilities and inconsistent availability as well as concern over low efficacy of medication in public facilities as important considerations. The research team met to consider the findings and defined 5 attributes for inclusion based on relevance to the research question and the decision context as well as independence across attributes (staff attitude, total waiting time, provider type, quality of clinical assessment, availability of free medication). Specifically, we did not include accessibility (transport time and/or cost) based on lower amenability to health system intervention or cost of services due to the focus of the research question on public health services. We prioritized availability of medication as the medication attribute of greatest relevance and potential for change within the public health services. Focus group findings supported independence of candidate attributes such as provider type and quality of clinical assessment, as respondents described provider role separately from clinical assessment, stating for example, "Even with nurse we are good to go as long as they have good knowledge, should be patient, should provide quality treatment. They should not be rude to us" (FGD 6: urban men). Participants also distinguished the examination process from receipt of medication in considering what high quality care meant to them. As one stated, "[Providers] don't examine us properly, they don't even touch us. The medicine is given only based on those symptoms that are explained by us" (FGD 3: rural men).

Table 2: Focus group findings on preferred attribute levels (N stickers assigned per attribute level)

**Attribute level**	**Votes (stickers)**
The providers are kind and respectful	103
Free medication for high-blood pressure is available	85
The providers assess or examine you carefully	78
The overall cost including transportation and any other payments is less than Rs 70	75
The provider is a doctor instead of a nurse	73
The wait time to a provider is short—about 30 minutes	60
The same providers are looking after you	47

© 2023 Leslie HH et al. *JAMA Network Open*.

The care will be provided by a team of providers	44
You will be referred to specialists at the hospital if needed.	25
The provider listens carefully to your questions and explains	23
You have privacy during the consultation	23
You can receive other non-hypertension services at the same facility	19
The most important tests for high blood pressure are available on site	17
The facility is clean and nice	15
Providers come to your community to measure your blood pressure and dispense drugs (e.g. camps, outreach activities)	15
Integrated services for my family are also available on site	12
There is a wellness area for yoga classes	2
Counselling on lifestyle modification for hypertension is offered in the facility	2
Peer support services are available on site	2

We defined attribute levels in order to reflect the current health service context, incorporate feasible improvements based on local policy standards, and avoid extreme levels that might dominate decision-making. For instance, although multiple participants described wait times up to a full day and occasional experiences of facility staff shouting at patients in line, we set the maximum level of waiting at 5 hours and the baseline for staff attitude as "not always courteous" to avoid overly aversive levels. Medication was defined as free in keeping with state policy for public health services and as always or not always available based on focus group discussions. Research team members (GRB, NDS, DR, SS) developed specific wording in both English and Kannada and worked with an artist to finalize a visual depiction to elucidate each level to ensure clarity and interpretation consistent with the intended meaning. All respondent versions included each attribute level a minimum of 7 times except for wait time levels, which each occurred 1-4 times per version (Table 3).

Table 3: Frequency of attribute levels by DCE version (16 alternatives per version)

		Version				
**Attribute**	**Level**	**1**	**2**	**3**	**4**	**5**
Staff courtesy	Not always	7	7	7	7	7
	Always	9	9	9	9	9
Wait time	15 minutes	3	2	3	1	3
	30 minutes	2	3	3	3	4
	1 hour	3	3	1	4	1
	2 hours	3	4	3	3	4
	3 hours	3	1	4	1	3
	5 hours	2	3	2	4	1
Personnel	Nurse	8	8	8	8	8
	Doctor	8	8	8	8	8
Careful assessment	Not always	8	8	8	8	8
	Always	8	8	8	8	8

© 2023 Leslie HH et al. *JAMA Network Open*.

Free medication availability	Not always	8	8	8	8	8
	Always	8	8	8	8	8

Validity Checks and Model Fitting

Methods

To evaluate the assignment of choice set versions to respondents, we compared population characteristics by version assigned using Chi square tests with significance level 0.05. To assess internal validity, we checked for incomplete responses and for ‘straight lining’ – selecting either alternative A or B in all choice tasks, which could indicate poor engagement with the DCE. We characterized dominance of attribute levels – frequency of respondents always selecting the option with a given level – overall and by location.

We assessed validity of the mixed logit model by fitting the model holding out the choice set seen by all respondents and predicting uptake based on attribute levels in the excluded scenario.

We tested functional form for wait time preferences by fitting alternative models – categorical, quadratic, and models with intercepts for shortest and longest wait times only – with other attributes included and evaluating model fit based on AIC and BIC statistics.

As a sensitivity analysis, we repeated the mixed logit model and the latent class analysis excluding individuals who skipped any choice task or who provided straight-line responses.

Results

Two DCE versions were distributed slightly unevenly (25% version 1 vs. 15% version 5); respondent characteristics were comparable across versions (Table 4). Twenty-two respondents (2.0%) skipped 1 choice task; 1 skipped 2 tasks. Eleven respondents (1.0%) provided ‘straight-line’ responses. All responses were retained for analysis; covariates used in analysis had no missing values.

Table 4: Respondent characteristics by DCE version

	Version 1 (N = 277)	Version 2 (N = 221)	Version 3 (N = 217)	Version 4 (N = 205)	Version 5 (N = 165)	Total (N = 1085)	Chi square test p-value
**Location**							0.860
Bengaluru	131 (47.3%)	113 (51.1%)	102 (47.0%)	100 (48.8%)	84 (50.9%)	530 (48.8%)	
Kolar	146 (52.7%)	108 (48.9%)	115 (53.0%)	105 (51.2%)	81 (49.1%)	555 (51.2%)	
**Gender**							0.843
Male	131 (47.3%)	100 (45.2%)	102 (47.0%)	96 (46.8%)	78 (47.3%)	507 (46.7%)	
Female	145 (52.3%)	119 (53.8%)	115 (53.0%)	107 (52.2%)	87 (52.7%)	573 (52.8%)	

© 2023 Leslie HH et al. *JAMA Network Open*.

Other	1 (0.4%)	2 (0.9%)	0 (0.0%)	2 (1.0%)	0 (0.0%)	5 (0.5%)	
**Highest education level completed**							0.611
No Formal schooling	63 (22.7%)	53 (24.0%)	60 (27.6%)	49 (23.9%)	36 (21.8%)	261 (24.1%)	
1st to 5th standard	67 (24.2%)	51 (23.1%)	41 (18.9%)	35 (17.1%)	36 (21.8%)	230 (21.2%)	
6th to 8th standard	58 (20.9%)	47 (21.3%)	44 (20.3%)	56 (27.3%)	43 (26.1%)	248 (22.9%)	
9th to 10th standard	54 (19.5%)	42 (19.0%)	51 (23.5%)	42 (20.5%)	32 (19.4%)	221 (20.4%)	
Diploma	31 (11.2%)	19 (8.6%)	17 (7.8%)	18 (8.8%)	12 (7.3%)	97 (8.9%)	
Degree / Graduate	4 (1.4%)	9 (4.1%)	4 (1.8%)	5 (2.4%)	6 (3.6%)	28 (2.6%)	
**Ever diagnosed with high blood pressure**							0.733
No	41 (14.8%)	35 (15.8%)	31 (14.3%)	29 (14.1%)	31 (18.8%)	167 (15.4%)	
Yes	236 (85.2%)	186 (84.2%)	186 (85.7%)	176 (85.9%)	134 (81.2%)	918 (84.6%)	
**Receiving HTN treatment at public primary facility (out of all respondents)**							0.255
No	160 (57.8%)	130 (58.8%)	136 (62.7%)	128 (62.4%)	112 (67.9%)	666 (61.4%)	
Yes	117 (42.2%)	91 (41.2%)	81 (37.3%)	77 (37.6%)	53 (32.1%)	419 (38.6%)	
**Prior treatment for hypertension (out of all respondents)**							0.645
No	54 (19.5%)	46 (20.8%)	35 (16.1%)	34 (16.6%)	33 (20.0%)	202 (18.6%)	
Yes	223 (80.5%)	175 (79.2%)	182 (83.9%)	171 (83.4%)	132 (80.0%)	883 (81.4%)	

In assessing response patterns, no single attribute showed substantial dominance: the attributes with strongest dominance patterns, careful assessment and availability of free medication, were always selected by 14.2% and 14.6% of respondents, respectively (Table 5). Respondents consistently selecting the option with availability of free medication were evenly distributed between study sites, while all other dominant respondents were more common in 1 location: those selecting based on wait time and courtesy were predominantly in Bengaluru Nagara (6.6% and 1.3% of urban respondents, respectively), while individuals consistently selecting based on seeing a doctor or receiving a careful assessment were nearly all in Kolar (15.0% and 27.4% of rural respondents, respectively).

The robustness check comparing predicted to observed choice found 64.5% predicted choice of alternative B compared to 62.5% observed in the scenario held out from model fitting, providing evidence of internal validity of the DCE and mixed logit modelling approach.

Table 5: Attribute dominance patterns by study location

	Bengaluru	Kolar	Total

© 2023 Leslie HH et al. *JAMA Network Open*.

	(N = 530)	(N = 555)	(N = 1085)	Fisher's exact test p-value
**Shorter wait time**				<0.001
Non-dominant	495 (93.4%)	546 (98.4%)	1041 (95.9%)	
Dominant	35 (6.6%)	9 (1.6%)	44 (4.1%)	
**Courtesy**				0.035
Non-dominant	523 (98.7%)	554 (99.8%)	1077 (99.3%)	
Dominant	7 (1.3%)	1 (0.2%)	8 (0.7%)	
**Doctor**				<0.001
Non-dominant	529 (99.8%)	472 (85.0%)	1001 (92.3%)	
Dominant	1 (0.2%)	83 (15.0%)	84 (7.7%)	
**Careful assessment**				<0.001
Non-dominant	528 (99.6%)	403 (72.6%)	931 (85.8%)	
Dominant	2 (0.4%)	152 (27.4%)	154 (14.2%)	
**Availability of free medication**				0.731
Non-dominant	455 (85.8%)	472 (85.0%)	927 (85.4%)	
Dominant	75 (14.2%)	83 (15.0%)	158 (14.6%)	

"Dominant" refers to respondents who always select the option with their preferred level of the indicated attribute, regardless of other attribute levels.

Assessment of the functional form of wait time is reported in Table 6: non-linear models did not clearly improve model fit. We maintained wait time as a continuous variable ranging from 15 minutes to 5 hours in subsequent analyses.

Table 6: Model fit statistics comparing forms of wait time

	Full (N=1085)	
	AIC	BIC
Continuous	**10233.6**	**10311.2**
Categorical	10266.6	10406.2
Quadratic	10237.4	10330.5
15-minute intercept	10298.7	10376.3
5-hour intercept	10277.7	10355.3

Findings from the overall and latent class models were unchanged in sensitivity analysis restricted to the 1051 respondents who completed all 8 choice tasks and selected alternative A and B at least once (Table 7).

Table 7: Sensitivity analysis excluding respondents with <8 completed choice tasks or straight-line responses (N=1051)

**Mixed logit analysis**		
Mean		
	β	95% CI
Wait time (hours)	-0.09	[-0.12,-0.05]
Staff are courteous (vs. not always courteous)	0.03	[-0.04,0.10]

© 2023 Leslie HH et al. *JAMA Network Open*.

Doctor (vs. nurse)	0.34	[0.24,0.43]
Staff assess patients carefully (vs. do not always assess patients carefully)	0.69	[0.57,0.80]
Free medication available (vs. not always available)	0.68	[0.56,0.80]
SD		
Wait time	0.26	[0.20,0.32]
Staff courtesy	0.01	[-0.10,0.11]
Doctor	0.98	[0.84,1.12]
Careful assessment	1.25	[1.11,1.40]
Free medication available	1.28	[1.13,1.43]
**Latent class analysis**	β	95% CI
Class 1		
Wait time	-2.90	[-4.64,-1.16]
Staff courtesy	-0.19	[-1.05,0.66]
Doctor	-0.97	[-2.47,0.52]
Careful assessment	1.14	[-0.50,2.78]
Free medication available	0.01	[-0.73,0.75]
Class2		
Wait time	-0.37	[-0.82,0.08]
Staff courtesy	-2.51	[-7.10,2.08]
Doctor	-1.23	[-2.60,0.14]
Careful assessment	6.60	[1.16,12.05]
Free medication available	-1.13	[-2.09,-0.16]
Class3		
Wait time	-0.08	[-0.37,0.22]
Staff courtesy	0.45	[-0.42,1.33]
Doctor	4.03	[2.77,5.30]
Careful assessment	0.59	[-0.34,1.52]
Free medication available	0.65	[-0.42,1.72]
Class4		
Wait time	0.15	[-0.16,0.46]
Staff courtesy	0.51	[-0.39,1.41]
Doctor	-0.28	[-1.00,0.45]
Careful assessment	0.34	[-0.42,1.09]
Free medication available	4.19	[3.12,5.26]
Class5		
Wait time	-0.01	[-0.04,0.01]
Staff courtesy	0.05	[-0.02,0.12]
Doctor	0.01	[-0.06,0.08]
Careful assessment	0.12	[0.05,0.19]

© 2023 Leslie HH et al. *JAMA Network Open*.

Free medication available	0.07	[-0.00,0.15]
Share1		
_cons	-2.25	[-2.58,-1.91]
Share2		
_cons	-1.18	[-1.38,-0.98]
Share3		
_cons	-1.69	[-1.95,-1.44]
Share4		
_cons	-1.12	[-1.33,-0.92]

© 2023 Leslie HH et al. *JAMA Network Open*.

eTable 1. Reflexivity Statement

	Question	
Study conceptualisation	1. How does this study address local research and policy priorities?<br>2. How were local researchers involved in study design?	The study was designed to provide insight into patient preferences for primary care in the context of the Health and Wellness Centre policy being rolled out in Karnataka. In this part of India, one in three adults above 30 years might have hypertension (HTN). It is estimated that nearly half of them miss being diagnosed and treated. Understanding the preferences of persons with Hypertension and their caregivers can inform the contextual changes in the policy of preventing complications due to HTN.<br><br>This study was one part of a project jointly led by Dr. Margaret Kruk (Harvard) and Dr. Dorairaj Prabhakaran (PHFI). The 9 co-authors include 4 currently or previously at Harvard and 5 from PHFI, all of whom were closely engaged throughout the study, from design through manuscript writing. During study design, the PHFI team led by Dr. Giridhar Babu in Bangalore was closely involved in DCE design, selecting appropriate attributes, the wording of attributes to suit the context and questionnaire development.
Research management	1. How has funding been used to support the local research team(s)?	Pilot award funding was divided 38% to Harvard and 62% to PHFI to support the project activities, including staff salaries, project costs, and data collection contract expenses.
Data acquisition and analysis	1. How are research staff who conducted data collection acknowledged?<br>2. How have members of the research partnership been	Co-author Nolita Saldanha conducted (focus groups) and oversaw data collection with support from co-authors Deepa Ravi and Dr. Babu. The

© 2023 Leslie HH et al. *JAMA Network Open*.

	Question	
	provided with access to study data?<br>3. How were data used to develop analytical skills within the partnership?	contracted data collection team is thanked in acknowledgments.<br><br>Study data are owned by PHFI and are available to all members of the research partnership.<br><br>Two formal training workshops were provided to all team members to build capacity in study methods during the course of the study: one on discrete choice experiments – design, administration, and analysis led by Dr. Kruk, and one on best practices for focus group conduct and analysis led by Dr. Turcotte-Tremblay. Analysis of focus groups was conducted jointly by research team members from both institutions (Harvard and PHFI).
Data interpretation	1. How have research partners collaborated in interpreting study data?	Results from focus groups were discussed on routine team calls in developing the DCE. Analysis of the DCE was shared in routine team calls to refine and interpret findings; all team members contributed to the written interpretation of findings.
Drafting and revising for intellectual content	1. How were research partners supported to develop writing skills?<br>2. How will research products be shared to address local needs?	All team members contributed to manuscript revisions.<br><br>Findings were summarized for a presentation to health system stakeholders in Karnataka; the meeting has been postponed due to the demands of the Covid pandemic and emerging variants on the local stakeholders.
Authorship	1. How is the leadership, contribution and ownership of this work by LMIC researchers recognised within the authorship?<br>2. How have early career researchers across the	The project as a whole was led by Dr Kruk, Dr Prabhakaran, Dr Babu, and Dr Leslie; this manuscript was led by Dr. Kruk and Dr. Leslie based on the

© 2023 Leslie HH et al. *JAMA Network Open*.

	Question	
	partnership been included within the authorship team?<br>3. How has gender balance been addressed within the authorship?	specific focus on the discrete choice experiment.<br><br>Early career researchers and co-authors Ms. Saldanha, Ms. Ravi, and Ms. Kapoor contributed in multiple ways to the project and manuscript, including study design, study administration, and supporting analysis.<br><br>5 of 9 co-authors are women.
Training	1. How has the project contributed to training of LMIC researchers?	Research team members participated in formal training workshops on discrete choice experiments and conducted focus groups in addition to informal capacity-building opportunities on data management and manuscript development.
Infrastructure	1. How has the project contributed to improvements in local infrastructure?	No contributions to date
Governance	1. What safeguarding procedures were used to protect local study participants and researchers?	Project leadership was shared by Dr. Kruk (Harvard) and Dr. Prabhakaran (PHFI); data are owned by PHFI and require Dr. Prabhakaran's permission for further use. All study procedures were reviewed by 3 ethical and research review panels in India to ensure local relevance and benefit. All protocols were adapted to the ongoing Covid-19 pandemic to ensure no added risk to any participants or researchers.

© 2023 Leslie HH et al. *JAMA Network Open*.

eFigure 1. Discrete Choice Experiment Introductory Script and Choice Card

*"*In this section I will show you 8 cards describing two possible health facilities that you could use to get health care for your high blood pressure in the future. These are new Health and Wellness Centers (HWCs) created by the government to improve health care available to communities. Imagine that you are deciding which of the centers you will choose for care for hypertension. To prevent health problems you will probably need to visit this health facility 3-4 times per year for checkups and laboratory tests and to obtain medicines.

As you are deciding, please know that both facilities are less than 1 hour away and a provider will be there when you arrive. Consultations at the centers will be free of charge and they can perform basic tests and provide medicine. If you need specialized care, they can refer you to a hospital.

Please tell us which of the two facilities you would prefer to go to for your care. There are no right or wrong answers to these questions and remember we will not share your information with anyone. We are only interested in learning about what is important to you about health facilities that will care for your high blood pressure."

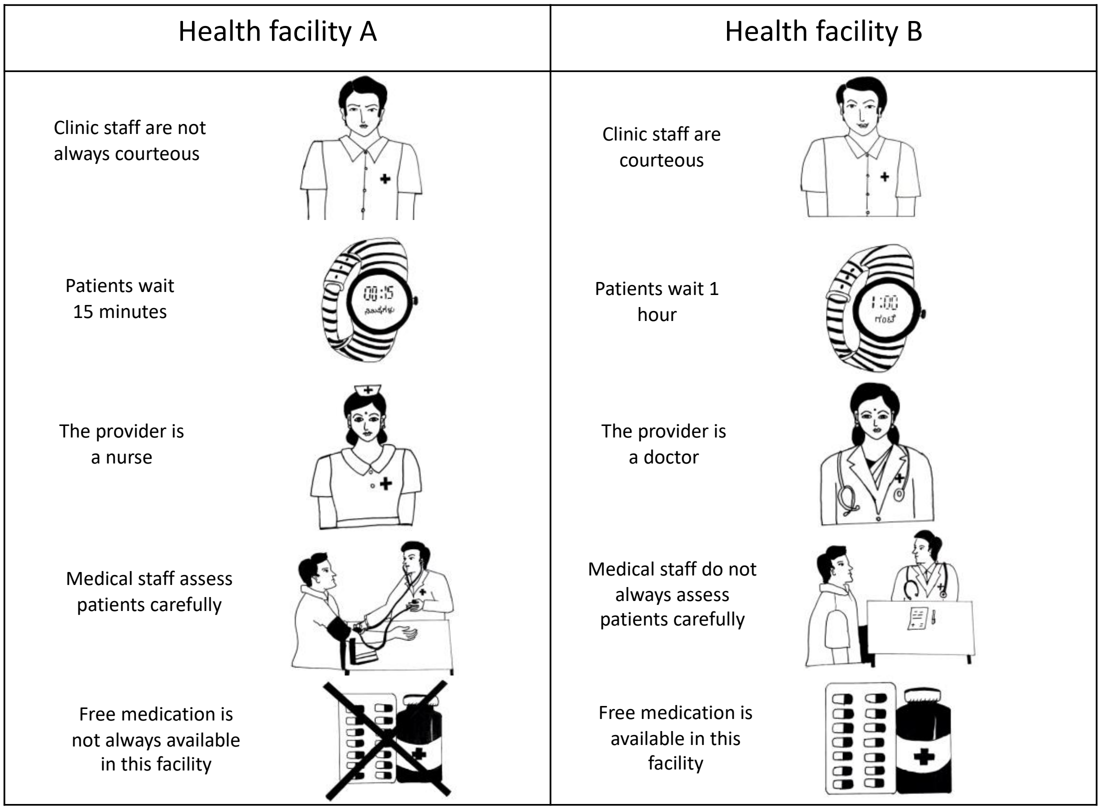

© 2023 Leslie HH et al. *JAMA Network Open*.

eFigure 2. Study Flowchart

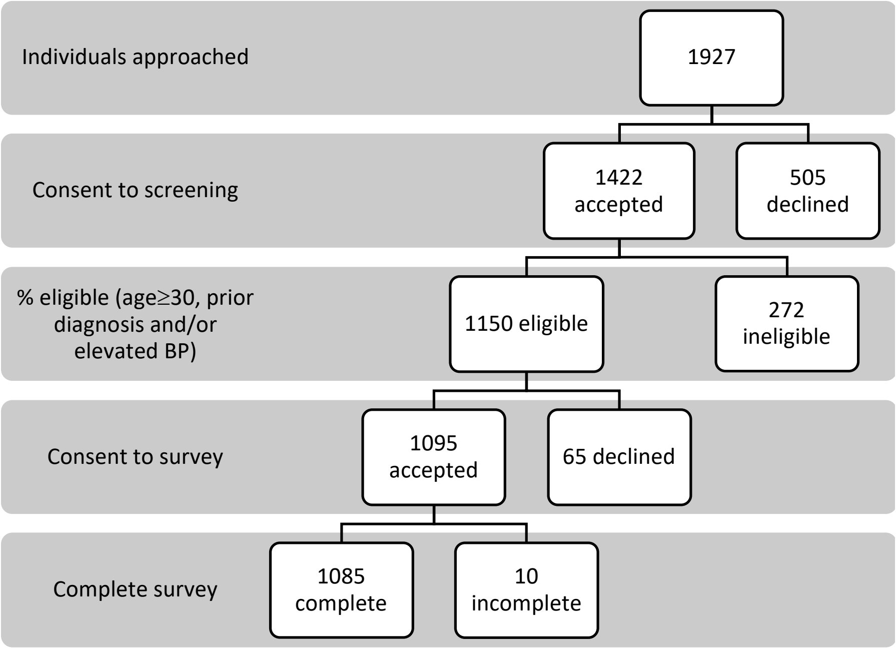

The main reason for declining was not wanting to spend the time on the survey

© 2023 Leslie HH et al. *JAMA Network Open*.

eFigure 3. Preference Shares for 5 Latent Classes

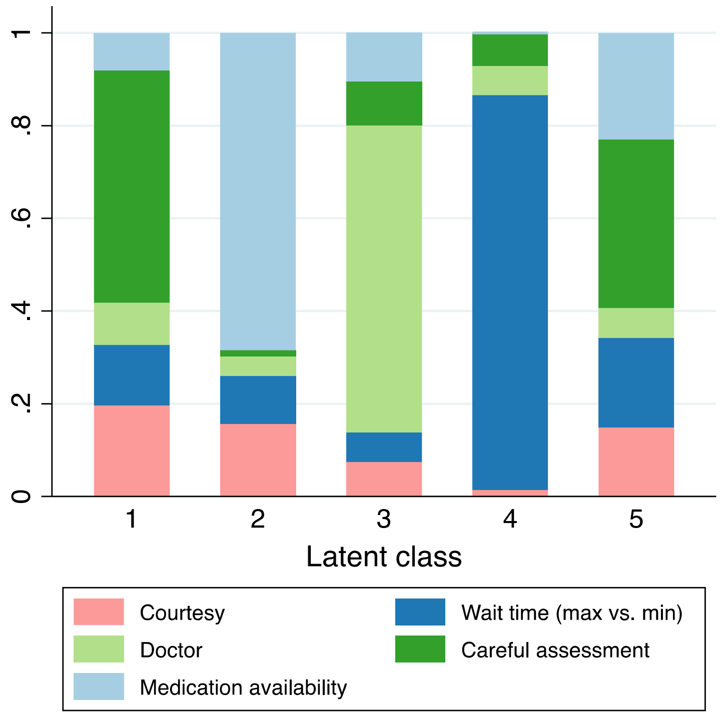

© 2023 Leslie HH et al. *JAMA Network Open*.

eTable 2. Latent Class Shares by Location

	Bengaluru (N = 530)	Kolar (N = 555)	Total (N = 1085)
**Class 1 share**			
Mean (SD)	0.03 (0.13)	0.28 (0.44)	0.16 (0.35)
**Class 2 share**			
Mean (SD)	0.18 (0.36)	0.16 (0.35)	0.17 (0.36)
**Class 3 share**			
Mean (SD)	0.02 (0.10)	0.17 (0.35)	0.09 (0.27)
**Class 4 share**			
Mean (SD)	0.09 (0.26)	0.02 (0.13)	0.05 (0.21)
**Class 5 share**			
Mean (SD)	0.69 (0.41)	0.37 (0.44)	0.52 (0.46)

Note: adjusted models including location could not be estimated due to the magnitude of class share differences by location.

© 2023 Leslie HH et al. *JAMA Network Open*.

eTable 3. Factors Associated With Latent Class Membership

	AOR	95% CI
Individual characteristics		
Reference class: Class 5		
**Share 1 (Class 1, careful assessment)**		
Gender: female	1.11	[0.76, 1.60]
Education: no formal education	**0.46**	**[0.27, 0.78]**
Not aware of hypertension	**3.54**	**[2.21, 5.66]**
**Share 2 (Class 2, free medication availability)**		
Gender: female	1.10	[0.76, 1.60]
Education: no formal education	**1.88**	**[1.27, 2.77]**
Not aware of hypertension	0.62	[0.31, 1.24]
**Share 3 (Class 3, doctor)**		
Gender: female	1.13	[0.69, 1.86]
Education: no formal education	0.51	[0.26, 1.01]
Not aware of hypertension	**4.01**	**[2.34, 6.87]**
**Share 4 (Class 4, wait time)**		
Gender: female	0.72	[0.39, 1.32]
Education: no formal education	**2.22**	**[1.19, 4.13]**
Not aware of hypertension	0.63	[0.21, 1.87]

Note: adjusted models including location could not be estimated due to the magnitude of class share differences by location.

© 2023 Leslie HH et al. *JAMA Network Open*.
