# Supplementary material for: Population Preferences for Primary Care Models for Hypertension in Karnataka, India
Source: JAMA Netw Open. 2023 Mar 14;6(3):e232937. doi: 10.1001/jamanetworkopen.2023.2937 (PMC10015308; doi:10.1001/jamanetworkopen.2023.2937)
Supplement: Supplement 2. — Data Sharing Statement [file jamanetwopen-e232937-s002.pdf]

Data Sharing Statement

Leslie. Population Preferences for Primary Care Models for Hypertension in Karnataka, India. *JAMA Netw Open*. Published March 14, 2023. doi:10.1001/jamanetworkopen.2023.2937

Data
Data available: Yes
Data types: Deidentified participant data
How to access data: A de-identified dataset used for this analysis will be made available on the Harvard Dataverse (https://dataverse.harvard.edu) at the time of publication.
When available: With publication

Supporting Documents
Document types: Statistical/analytic code
How to access documents: Statistical code is available from the corresponding author (hannah.leslie@ucsf.edu)
When available: With publication

Additional Information
Who can access the data: Anyone requesting the data
Types of analyses: For any purpose
Mechanisms of data availability: With investigator support
